# Study on Health of Older People in Germany (Gesundheit 65+): objectives, design and implementation

**DOI:** 10.25646/11666

**Published:** 2023-09-20

**Authors:** Judith Fuchs, Beate Gaertner, Hanna Perlitz, Tim Kuttig, Annett Klingner, Jens Baumert, Antje Hüther, Ronny Kuhnert, Julia Wolff, Christa Scheidt-Nave, the study group Gesundheit 65+

**Affiliations:** Robert Koch Institute, Berlin, Germany Department of Epidemiology and Health Monitoring

**Keywords:** HEALTH, OLD AGE, LONGITUDINAL STUDY, POPULATION-BASED, GERMANY, COVID-19, HEALTH SURVEY

## Abstract

**Background:**

The longitudinal population-based study Gesundheit 65+ aimed to close data gaps on health and well-being of older adults in Germany in times of the COVID-19 pandemic.

**Methods:**

The target population comprised persons 65 years and older permanently residing in Germany and with sufficient German language skills. Proxy interviews were possible and consent from legal representatives was obtained as necessary in order to enable participation of physically or cognitively impaired persons. A two-stage sampling process, was used to draw 128 primary sample points (PSUs) and within these PSUs sex- and age-stratified random samples were drawn from population registries. A mixed-mode design was applied to contact the study population and for data collection. Data were collected between June 2021 and April 2023. Participants were surveyed a total of four times at intervals of four months. At month 12 participants were offered a home visit including a non-invasive examination. Data on all-cause mortality and information on neighborhood social and built environment as well as health insurance data will be linked to primarily collected data at the individual level.

**Discussion:**

Results will inform health politicians and other stakeholders in the care system on health and health care needs of older people in Germany.

## 1. Background

As in many other countries, the proportion of older and very old people in the population in Germany will continue to rise due to increasing life expectancy and low birth rates [1]. As of 2022 18.6 million people living in Germany are 65 years of age and older, including 6.1 million who are 80 years and older [2]. With increasing age, the probability of illness and the decline in physical and cognitive function increases. The majority of older people today are able to actively participate in social life. However, at a given chronological age the spectrum of health and functional state in older age ranges from completely unrestricted, independent and integrated in social life to very severely restricted and permanently dependent on support and nursing care. Apart from genetic factors, social and environmental determinants of health largely contribute to the heterogeneity in health and health-related functional limitations among older adults [3, 4].

The COVID-19 pandemic posed major challenges to health care systems worldwide. Older adults, in particular persons 80 years of age and older, and residents of long-term nursing care facilities were at particularly high risk of severe or fatal COVID-19 in Germany as in other countries [5–8]. In addition, containment measures such as contact restrictions and changes in access and use of health care services affected the entire population, but had different impacts depending on age [9, e.g. 10, 11–14]. Information representative of the population aged 65 years and older is limited.

As part of the nationwide health monitoring in Germany conducted by the Robert Koch Institute (RKI), Gesundheit 65+ was designed to provide current insight into health and well-being of the population aged 65 years and older in times of the COVID-19 pandemic. The study puts a focus on including functionally impaired old and very old people. In contrast to previous health monitoring studies of the RKI [15, 16], the present study therefore applies a previously tested study protocol aimed to include older persons with physical and cognitive impairments [17, 18].

Health in older age was assessed based on primary interview and measurement data collected during the COVID-19 pandemic, which can be linked to data from additional sources like statutory health insurance data or geographic information systems. In detail, analyses of these data will provide insight on:

1. health status and well-being based on self-reported data from the cross-sectional baseline survey,
2. changes in subjective, physical and mental health, individual and social health determinants and utilization of health care services based on self-reported longitudinal data and
3. core indicators of objective physical health as well as physical and cognitive functional status based on cross-sectional data including standardized tests and measurements for groups of older adults with special health care and social support needs.

## 2. Methods

### 2.1 Study design

#### Study design and sample

**Figure 1:**
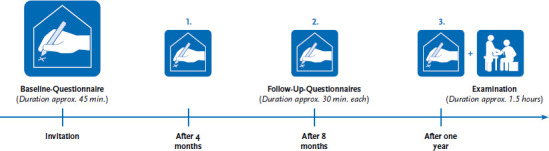
Data collection in Gesundheit 65+ Source: Gesundheit 65+, own description

Gesundheit 65+ is a population-based longitudinal epidemiological study to provide representative data on the health situation of people aged 65 years and older in Germany. A longitudinal survey design was chosen to closely map changes in well-being, health and functional status as well as changes in medical care services and health care services utilization during the course of the COVID-19 pandemic. Data were collected between June 2021 to April 2023 and included a baseline interview survey, which was completed between June 2021 and April 2022, as well as follow-up surveys four, eight and 12 months after the baseline survey (Figure 1). Participants were visited at home for a physical examination and a drug interview at the time of the final follow-up.

The study was funded by the Federal Ministry of Health Germany (Grant No: ZMVI1-2518FSB410) and approved by the ethics committee at the Berlin Chamber of Physicians (German: Berliner Ärztekammer, Eth-50/19) and the data protection officer of the RKI. It was conducted in compliance with the data protection provisions set out in the EU General Data Protection Regulation (GDPR) and the Federal Data Protection Act (BDSG). Our data protection concept included the use of register-based information and the linkage with publicly available data for non-responder analyses and the search for a telephone number to contact individuals via telephone.

Prior to study enrollment, study participants or their legal representatives provided written informed consent to participate in the study. Participation by means of an online baseline questionnaire was an exception; here, the invited person could also give consent online. In addition, consent could be given at this time for (a) geocoding of the home address for linkage to social and environmental health determinants of the living environment and (b) vital status follow-ups at residential population registration offices (i.e., their survival time). Participants or their legal representatives were informed that the study was voluntary and they could choose to withdraw from the study or any parts of it at any time. Written consent from the invitees or their legal representatives was also required for participation in a non-responder questionnaire/interview at baseline. Oral consent was only possible if the invited individual consented to a telephone non-responder interview.

Prior to health examinations on completion of the study by home visits, participants or legal representatives provided a separate written informed consent. Written informed consent to the linkage of individual ambulatory statutory health care data was also obtained at the home visit.

#### Inclusion and exclusion criteria

The target population comprised persons 65 years and older permanently residing in Germany. We excluded persons not able to understand German language, as well as individuals who had died/moved before the field period started or were untraceable.

Persons who, e.g were not able to provide information about their health and to participate in the surveys themselves, could participate by asking a proxy. Persons unable to provide written informed consent were able to participate based on written informed consent provided by their legal representative.

#### Sampling procedure

**Figure 2:**
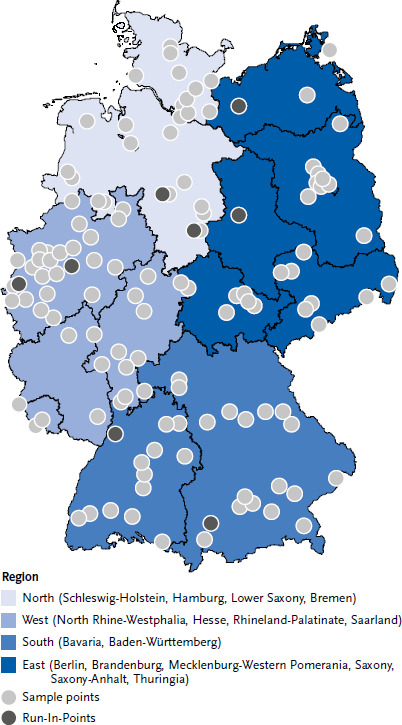
Primary sampling units of Gesundheit 65+ Source: Gesundheit 65+, own description

A two-stage stratified cluster sampling procedure was applied analogous to the previous population-based RKI health surveys DEGS1 und GEDA 2014/2015-EHIS [19, 20]. In the first stage, 120 primary sampling units (PSUs) were randomly drawn from all municipalities in Germany in collaboration with the Leibniz Institute for the Social Sciences (GESIS), Mannheim, Germany. Eight additional PSUs were drawn to be used for a run-in period. All 128 selected PSUs are displayed in Figure 2. Random selection of PSUs was stratified based on region and the BIK-10 classification, a regional classification system for Germany [21], in order to adequately represent low-population municipalities and to allow for prevalence estimates in four major regions in Germany. The following regions were considered: North (Schleswig-Holstein, Hamburg, Lower Saxony, Bremen), South (Bavaria, Baden-Württemberg), West (North Rhine-Westphalia, Hesse, Rhineland-Palatinate, Saarland), and East (Berlin, Brandenburg, Mecklenburg-Western Pomerania, Saxony, Saxony-Anhalt, Thuringia). Municipalities with very large populations (e.g. Berlin) are represented by several PSUs. Communities with few inhabitants aged 65+ years were grouped with neighboring small communities belonging to the same stratification cell. In the second stage, within PSUs, sex- and age-stratified random samples of the population aged 65 years and older were then drawn from local population registers applying unrestricted random selection. Stratification by age considered two age groups (65 – 79, 80 + years).

#### Statistical precision and power

To estimate the precision of descriptive prevalence estimates, a corresponding interval of less than 10 % of the expected prevalence was used for the width of the confidence interval.

Power calculations showed that a total of 128 sample points and an overall sample size of 2,700 participants would permit estimation of prevalence rate stratified by sex and age group (65 – 79, 80 + years) at the final follow-up contact (i.e. the examination) with sufficient precision. This calculation assumed comparable numbers of participants by strata and PSU at baseline and, based on previous experience [18], a 32.5 % loss from baseline participation to the last follow-up. Cluster sampling underestimates total variability and hence estimation error compared to simple random sampling, because individuals within PSUs are more likely to share study characteristics than individuals directly sampled from the population. The increase in estimation error is quantified by the ‘design effect’ which can be assumed as 1.5 (i.e. 50 % more participants are needed, compared to a one-stage sampling design) for the age group 65 and older based on own unpublished analyses of previous RKI population-based health surveys. Due to financial constraints we had to cut down the possible number of examinations per PSU leading to a total number of 1,500 possible examinations at the last follow-up. We repeated our power calculation as described above. Results were similar to the previous calculations. However, confidence intervals for prevalences of 30 % and more were slightly wider; the criterion of interval width less than 10 % was slightly exceeded here.

### 2.2 Conducting the survey

#### Field work strategy (Touring schedule)

In order to avoid systematic (e.g. seasonal or COVID-19-pandemic-related) effects on data collection, the 128 PSUs were randomly grouped into 32 routes. Including a total of four PSUs per route, one PSU was randomly assigned from each of the four regions in Germany and invitations were sent out successively on a route-by-route basis. The sequence of inviting and visiting PSUs for enrollment of study participants into the baseline survey was set in a touring schedule before starting the invitation process.

#### Baseline recruitment

**Figure 3:**
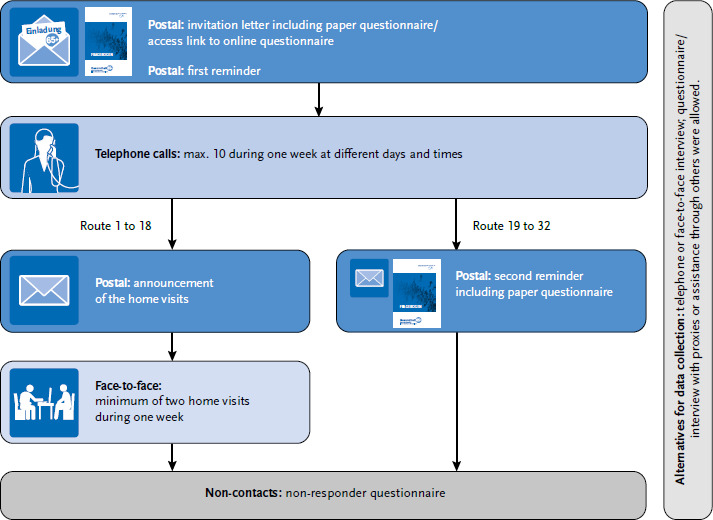
Sequential contact design at baseline Source: Gesundheit 65+, own description

A previously developed and tested sequential mixed-mode design [17] was applied to contact the sampled individuals for study participation (Figure 3). In a first step and in addition to a brief personalized invitation letter, the invitees received an information brochure, the baseline paper questionnaire, a consent form, a prepaid envelope for returning the questionnaire, a response form and a small unconditional incentive (pack of flower seeds). The brochure informed invitees on study design (i.e. participation options, planned follow-ups, examination, funding and data protection. After two weeks without response to the invitation, individuals received a postal reminder. The invitation as well as the reminder letter provided a link and a personalized password to an online questionnaire and the offer of a telephone interview as additional options for participation. One week after there was no response to the reminder, trained research assistants contacted the sampled individuals via telephone in order to inform about the study and the interview options, if a telephone number was available. About 2.5 weeks later, research assistants traveled to the PSUs and tried to contact invitees if no contact or decision on participation had been achieved. The research assistants informed about the study, provided information material (if necessary for other involved persons such as legal representatives or nursing home managers), helped with the questionnaire or offered a face-to-face interview. Four research assistants visited four PSUs per week (one PSU per assistant), in the following week four other research assistants visited the next four PSUs. It took approximately nine months to complete the baseline survey visiting all 32 routes. Home visits were announced in advance by mail including photographs of the research assistants (50 % women; age range: 41 – 67 years; mean age 54.7 years) who conducted these visits. For identification purposes, research assistants used an official RKI employee identification card. Home visits were stopped after route 18 in November 2021 due to the renewed COVID-19 surge at that time. Instead, from route 19 onwards, sampled individuals received a second postal reminder including the baseline questionnaire and a prepaid envelope. Women 80 years of age and older had turned out to be particularly hard to reach. From route 19 onwards, this group was therefore given another unconditional incentive within the second reminder letter (i.e. monetary voucher of 5 or 10 or 20 €).

The study protocol permitted assisted and proxy participation, i.e. study participants could get help through others such as family or friends with answering the questionnaire/interview. If the participants were not able to take part themselves due to cognitive or physical limitations, they or their legal representatives could authorize other individuals to answer the questionnaire/interview for them (so-called proxy interview).

If invitees or others had further questions at any point, they could contact the study’s toll-free service number from Monday to Friday between 9 to 12 a.m. and 2 to 5 p.m. or leave their concerns on an answering machine outside these hours, send their questions via email or could find additional information on the research project’s website. Invitees who did not wish to participate could indicate this in writing, by email, by phone or during the home visit.

Individuals who declined to participate as well as non-contacts were asked to answer a short non-responder questionnaire either at the time of study refusal or per mail approximately seven weeks after the initial invitation. In this questionnaire, essential sociodemographic and health-related characteristics were assessed to enable analyses of non-response and selection bias. Apart from sending back the filled-in questionnaire in a prepaid enclosed envelope, the questionnaire could also be answered by telephone or face-to-face interview during the home visit if desired.

#### Public relations work

To raise awareness of the Gesundheit 65+ study, a short study name and a logo were generated and used on all print and online materials. The launch of the study was announced with a national press release (in close cooperation with the RKI press office). In addition, at each of the 128 PSUs, mayors or municipal councils as well as the local press were informed about the study approximately two weeks before the invitation letters were sent out. The purpose of this local and regional public relations work was to increase awareness and trustworthiness of the study.

A detailed study website in German (www.rki.de/gesundheit65plus) informs invitees, participants and interested parties about the aims of the study and provides detailed information about the exact process, participation, data protection and study results.

#### Re-contacting for the follow-up surveys and the examination

All baseline participants who had not withdrawn their consent for re-contact and who were not deceased according to prior information (e.g. from relatives) were eligible for follow-up contacts. This means that individuals who had not participated in the follow-up surveys and had not completely declined further study participation were also invited again to the next follow-up survey. An address inquiry was conducted at the respective registration office for persons for whom the invitation letter for a follow-up was returned as undeliverable or who were not reached during the entire re-contacting process, and who had previously consented to this. Any address changes or deaths that became known as a result were taken into account for further re-contacting.

The same field work strategy as for baseline recruitment was used regarding routes and sequence of all invitations. All follow-up contacts were initiated by mail. The mailing included a personalized invitation letter (containing a link and a personalized password to an online version and the offer of a telephone interview), the paper follow-up questionnaire, and a small unconditional incentive (e.g. a pen or four bookmarks). Non-responders were contacted by telephone two to three weeks after invitation and, if no contact or decision on participation was achieved, were sent a reminder letter. Invitees who did not wish to participate could indicate this in writing, by email, or by telephone. In case of a refusal by phone, persons were asked to indicate the reasons for non-participation. Persons stating non-participation by mail and non-responders to follow-up contacts received a letter asking them to indicate the reasons for non-participation according to a checklist via prepaid return mail.

This procedure was the same for all follow-ups. As part of the invitation to the third follow-up, participants were invited to a brief health examination survey during a home visit. An additional brochure informed about the content and procedure of the examination as well as data protection in text and pictures.

From route 24 onwards in order to mitigate effects of self-selection for participation in the examination, only a reduced number of men aged 80 and over was invited at random for the examination. Up to this point, this group of persons had been reached better than women for the examination and was disproportionately represented in the sample compared to younger men due to the stratified sampling procedure.

### 2.3 Data collection methods, contents and data linkages

#### Content of questionnaires/interviews

In order to lower participation barriers for very old or functionally impaired persons participation efforts were kept as low as possible. We used large print documents to mitigate barriers due to sensory limitations. A mixed-mode data collection design offered a questionnaire on paper (also available as an online questionnaire) or an interview mode. Participants could be assisted by a third person at any time and proxy participation was allowed. Due to data linkage (e.g. linkage with ambulatory health care data) content of health questionnaires/interviews could be focused on health indicators requiring self-reported information. Where possible, questions and instruments were selected from other studies currently conducted by the RKI to enable comparative analyses.

The baseline questionnaire included but was not limited to essential health concepts for older and very old people (e.g. physical and cognitive functioning, mobility, falls, nursing care needs and assistance with basic or instrumental activities of daily living). The selection of indicators was based on a previously developed set of health indicators for the population 65 years and older [22]. In addition, the questionnaire covered the main topics of health: self-perceived health, and health-related quality of life, physical and mental health status, health-related behaviors, utilization of health care and preventive services, social and environmental determinants of health. A special focus was put on the direct and indirect health impacts of the COVID-19 pandemic. The follow-up questionnaires contained selected questions from the baseline questionnaire in order to permit tracking changes in physical and mental health (e.g. self-perceived health, falls, depressive symptoms), health-related behaviors as well as social and psychological determinants of physical and mental health (e.g. social support and loneliness). Table 1 provides an overview of the content of the questionnaires.

#### Selection of measurements and tests

**Figure 4:**
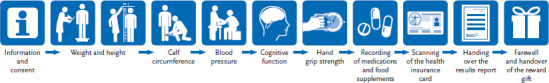
Course of measurements, tests and recordings in Gesundheit 65+ Source: Gesundheit 65+, own description

Parallel to the final follow-up questionnaire, study participants were invited to take part in a home visit examination with an average duration of 1.5 hours. This enabled even people with limited mobility to participate without great effort. The standardized examinations and tests used have been tested in previous surveys [16, 18]. Figure 4 provides an overview of the course of the home visit and Table 2 provides an overview of the tests and instruments used. After completion of the examination, participants received written information on their test results and a small gift (material value approx. 5 €).

#### Data linkage

Based on the participants’ or legal representatives’ informed written consent linkage of primary data collected in Gesundheit 65+ to health-related data from various other sources will be possible. Data from external sources include:

1. vital status information obtained from residential population registration offices over a maximum of 20 years to analyze patterns and determinants of all-cause mortality,
2. social and built residential environment characteristics obtained through geocoding of the participants’ addresses at baseline in order to enable analyses of social and environmental determinants of health, e.g. social area deprivation, health care infrastructure, exposures to noise and air pollution, and
3. ambulatory statutory health care data will be linked by an independent trust office for analysis of self-reported health data in conjunction with documented medical diagnoses and health care services provision in collaboration with the Central Research Institute of Ambulatory Health Care in Germany.

#### Baseline non-responder questionnaire

A non-responder analysis is essential to assess selection bias in Gesundheit 65+. Therefore, a brief non-responder questionnaire or interview was offered to all baseline non-participants. Proxy participation, e.g. by relatives or caregivers, was possible, if the invitee or her/his legal representative gave consent.

The non-responder questionnaire/interview contained selected questions from the baseline questionnaire/interview as indicated in Table 1. In addition, reasons of non-participation were assessed according to a checklist.

### 2.4 Data processing and expected results

#### Quality assurance

A quality assurance (QA) concept was developed for this study. The QA concept included and defined the following: appointment of topic-based Quality Assurance Officers (QAO), responsibilities for several processes, communication structures within and outside the project, measures such as trainings, Standard Operating Procedures (SOPs), checklists, site visits, and documentation of the measures as well as evaluation of their effectiveness. All measures were supervised by a study-independent QA team established at the RKI.

SOPs were prepared for all steps of the data collection process including checklists for standardized interviews and examinations. Supervised training periods of several weeks were scheduled for study personnel before the start of baseline invitations and the follow-up examination. Training days were scheduled prior to starting first and second follow-up contacts in order to teach the specifics of contacting (e.g. changed order of contact modes, see Re-contacting for the follow-up surveys and the examination) and contents of the data collection (see Table 1). Adherence to standardization in interviewing and examination according to SOPs/checklists were verified in advance of data collection and through site visits. If required, additional individual trainings were conducted. In addition, process data, e.g. on the number and mode of contact attempts per wave, were continuously evaluated and retrainings were conducted in case of deviations according to predefined rules.

Baseline and follow-ups started with a run-in period, i.e. all procedures were tested on the first two routes of eight sample points and could be adjusted as needed.

#### Data management plan

In parallel to the data collection the data management was carried out. The mixed-mode data collection design required different methods for data management that have already been used in previous RKI studies and include the handling of original data, raw data, data correction and finally the provision of analysis datasets. QA actions were implemented into every step of data processing and staff training.

Paper questionnaires were scanned, saved, digitally archived and verified by trained RKI staff. The quality of data entry was assessed via second entry of 10 % of the paper questionnaires. In computer-assisted web interviews (i.e. online questionnaire), in computer-assisted personal interviews (i.e. medication review during home visit examination) and in computer-assisted telephone interviews interviewers entered data directly into a software where plausibility checks were integrated by setting valid ranges and logical skips through filters. During baseline home visits or examination visits, face-to-face interviews were conducted paper-based and were entered electronically afterwards as described. Examination data was recorded with computer-assistance or electronically via the specific measurement devices (i.e. blood pressure).

All these raw datasets were combined into one inspection data set. Subsequently, this was used for data editing with a software syntax to obtain a research dataset. All changes on the datasets were double checked, documented and can be corrected as necessary. The raw data contains only corrections for data entry errors and is kept separate from the fully processed data record. To ensure a uniform procedure during data checking and correcting, general and additional study-specific SOPs were used for work processes in data management and QA. In addition, QA included checking for data completeness at the individual level, extreme values, missing values, compliance with filter questions etc. as well as for consistency and plausibility, accuracy and correctness in terms of content. Responses to open-ended questions were coded, aggregated variables and instruments were created. The research dataset includes all variables that have passed the data privacy check and have been cleared for release by the QA study management. This data is provided with supplementary imputed variables, weighting variables and detailed data documentation (data description, codebooks etc.).

#### Statistical analyses

We developed a statistical analysis plan for stepwise analyses of data based on methodological and public health priorities. This includes the cross-sectional analysis of health data collected during the baseline survey with a particular focus on previously defined core health indicators for older persons [22], longitudinal analyses of intraindividual changes in health status, well-being and analyses of the examination data.

For the statistical analyses a weighting factor was computed in order to correct study results for deviations of the study population from the target population of people 65 years of age and older in Germany as of 31 December 2020 with regard to sex, age, region and community size according to BIK-10 regional classification system for Germany [21]. In addition, the weighting factor will consider deviations in educational level according to the International Standard Classification of Education ISCED 2011 compared to data representative of the German resident population based on the German microcensus 2018 [23]. For the follow-ups, this weighting factor will be multiplied by the inverse of the estimated re-participation probability using appropriate regression methods and predictors to account for loss to follow-up. Descriptive statistics as well as multivariable logistic and other regression analyses for complex survey samples will be applied throughout the analyses of results.

## 3. Discussion

Gesundheit 65+ is the first nationwide population-based health study of older adults in Germany, which is specifically designed to include older and very old people as well as people with health limitations. Including older people in ongoing national health monitoring is crucial to evidence-informed health policy planning and the implementation and evaluation of national health goals, but also challenging [24]. Gesundheit 65+ applied a previously developed and tested mixed-mode contact and data collection design [17, 22, 25]. The intention was to lower participation barriers for older and impaired people who are often excluded or underrepresented in health surveys [17, 26, 27]. Adults 65 years of age and older are heterogeneous with regard to health and functional status, health-related behavior, coping with multimorbidity, social support and participation, and medical and nursing care needs. Reliable and actionable data on health of older people can only be obtained if people aged 65 and over provide information on their health, living situation and needs [e.g. 28].

Gesundheit 65+ aimed to collect population-representative data that cannot be obtained from any other data sources. In addition to cross-sectional health information obtained during the baseline health interview survey and the health examination as part of the home visit 12 months after baseline, follow-up interviews permit longitudinal analyses of intraindividual variability in health status, health resources and well-being of older adults in Germany in times of the COVID-19 pandemic. Combined with information from routine data, official health statistics and other data linkages, the results will support health policy planning and implementation research to improve the health and well-being of older people in Germany, and not least will contribute to future pandemic preparedness.

### 3.1 Strenghts and Limitations

The strengths of the study are as follows: First, we applied probability sampling and adapted the study design to enable participation of functionally impaired older persons. In order to keep participation barriers and selection bias as low as possible, we allowed assisted and proxy participation in the health interview part of the study and limited exclusion criteria, i.e. to persons with insufficient German language skills. Individuals having a legal representative or who were living in nursing homes were therefore included in our study. As can be seen from the first results of the Gesundheit 65+ study presented in the same issue of the Journal of Health Monitoring, only 307 people had to be excluded from participation in the study due to this approach. Furthermore, a participation rate of 30.9 % was achieved, which is high for this particular age group. Future analyses of the methodology will have to show to what extent the complex recruitment procedure was able to compensate for sampling biases, whether survey data from different survey modes are comparable, and whether there are biases in the examination sample due to the limited offering. Second, our study was carried out in accordance with our QA concept and highly standardized. Members of our research team were extensively trained and continuously supervised during all steps starting with study recruitment to data collection and the data set production.

We also see some challenges and limitations in the realization of this study. First, as other health researchers have already described, our study was affected and challenged by the COVID-19 pandemic [29]. The start of field work was originally planned for March 2020 and had to be canceled due to the first lockdown in Germany. When the study was restarted, the study questionnaire needed to be adapted to the challenges of the COVID-19 pandemic by including questions on infection and vaccination as well as indirect impacts of the pandemic on well-being, health status and health resources. However, this challenge also gave us the opportunity to collect population-based data on changes in health and functional status, health behavior and social health determinants during the pandemic. Due to the pandemic and the following contact restrictions it was necessary to temporarily adapt the study design by focusing on health interview data including a baseline survey and follow-up contacts and postponing the health examination part. Field work started in June 2021. Baseline recruitment required personal contacts to persons preferring face-to-face interviews and to persons without reaction to written invitations and not contactable by telephone. This was accompanied by high demands on the hygiene concept of the study, which made personal visits much more strenuous for our study team. Despite the hygiene concept, the feasibility of the study (especially the postponed examination) was uncertain throughout the whole study period. For example, face-to-face study recruitment had to be discontinued in November 2021 for the remaining baseline field period, when the SARS-CoV-2 Omicron variant became prevalent, posing a high risk of infection and severe illness to older persons. Future non-response analyses will have to show to what extent this had an impact on participant recruitment (e.g. on the composition of the sample).

Second, our study design is cost intensive. Conducting home visits to older people in Germany over an extended period of time required a large team of research assistants with high levels of commitment and motivation. Home visits are essential to enable older persons with health problems and those who depend on assistance to participate in research studies. However, they also increase the time required for data collection, as study staff must travel to and from the participants’ home addresses and set up and take down study equipment. Not all participants prefer a home visit to a study center examination. Examination in a conveniently located study center would therefore be a desirable complementary option to offer, which could be considered in a follow-up study. In the context of our study, however, renting a suitable study site in 128 different cities or municipalities in Germany turned out to be too complex.

Third, even with all the offerings of our study design, very severely impaired people, in particular nursing home residents, remain a hard-to-reach group [30], since nursing home staff and often also legal representatives need to be involved. The COVID-19 pandemic put additional barriers to the inclusion of older persons living in nursing homes. External visitors were granted only limited access even when providing proof of COVID-19 vaccination or a negative rapid test. Currently, roughly 16 % of the population aged 65 years and over in Germany receive some degree of nursing care, among these more than one fourth in long-term nursing facilities [31]. In order to routinely include older persons living in long-term care facilities into the national health monitoring, future studies will have to develop appropriate study designs tailored to the specific needs of long-term nursing care residents. At the same time, these studies need to be conducted in parallel to studies of health of older persons in private households in order to provide a full picture [28, 32].

Fourth, the EU General Data Protection Regulation [33] has increased the data protection requirements for scientific studies. In some cases, this may lead to greater uncertainty among invitees, e.g. due to lengthy and detailed data protection statements. Therefore, the comprehensive study brochure includes a passage on data protection in simple language and the complete data protection was added at the end. It was not possible to create a data protection statement that provides comprehensive data protection information to invitees and at the same time is written in simple language that can also be understood by people with reading or cognitive limitations. Future research or national and international research societies will have to show how this can be improved.

Finally, public relations for a nationwide study is an important part of creating trust and informing the public about the study. However, contacting mayors and local media outlets was time-consuming, as contacts in 128 different locations had to be researched and notified. It is not possible to evaluate the extent to which we succeeded in increasing trust and willingness to participate among invitees. In addition, the RKI, as the national public health institute in Germany, was in a strong public focus during the management of the COVID-19 pandemic at that time. This could have led to both a higher and a lower willingness to participate among invitees.

### 3.2 Perspectives

To conclude, Gesundheit 65+ provides a comprehensive dataset on health and well-being of older people in Germany during the COVID-19 pandemic, which will be available as a scientific use file for other interested researchers on request and as part of a research collaboration (expected end of 2024). Study results will be presented to stakeholders in the German health care system, to the scientific community based on conference contributions and publications, and to the public via website information as well as a visualized lay briefing on study results for study participants. To provide continuous health reporting and policy guidance in aging populations, it is necessary to establish a national public health surveillance system for the population aged 65 years and older. For this purpose, a panel is currently being set up at the RKI, into which the data collection of the people aged 65 years and older will be integrated, including regular health examination surveys in Germany. In addition, there are also efforts to establish health surveillance systems for older people living in long-term care facilities in Germany [34].

## Key statement

- Gesundheit 65+ integrates the population aged 65 and older without an upper age limit into the RKI’s nationwide examination surveys.
- The longitudinal study consists of a baseline questionnaire, three follow-up questionnaires and a home visit examination.
- Various options were offered to enable physically or cognitively impaired individuals to participate.
- Gesundheit 65+ collects information on subjective, objective and social aspects of the health of older people with the possibility of linking external data.
- Gesundheit 65+ contributes to evidence-based policy advice and public health research in a society of demographic change.

## Figures and Tables

**Table 1:** Overview over content of baseline, follow-up and non-responder questionnaires/interviews Source: Gesundheit 65+

Constructs, Measurement	Instrument/Source	Assessment timepoint	Non-responder questionnaire
**Health status**			
Self-perceived health	MEHM [35, 36]	T0 – T3	x
Change of self-perceived health	Self-developed close-ended question	T1 – T3	
Chronic disease	MEHM [35, 36]	T0	x
List of age relevant chronic diseases or conditions (past 12 month)	from EHIS [37]	T0, T3^a^	
Cancer (lifetime) and cancer treatment (past 12 months)	Self-developed close-ended question	T0	
Other chronic diseases with current treatment or impairment of daily life	Self-developed open-ended question	T0, T3	
Pain intensity in the last 4 weeks, duration	SF-36 Health Survey [38], Deutscher Schmerzfragebogen (German Pain Questionnaire) [39]	T0, T3	
Appetite and loss of appetite (past 12 month)	Adapted from Health ABC study [40, 41]	T0, T3	
(Unintentional) weight loss (past 12 month)	According to Fried et al. [42]	T0, T3	
**COVID-19 related questions**			
Infection	Self-developed close-ended question	T0 – T3^b^	x
Treatment	Self-developed close-ended question	T0 – T3^b^	
Vaccination, intention	Self-developed close-ended question	T0 – T3^b^	x (only vaccination)
Adherence to containment measures	Self-developed close-ended question	T0	
Unmet support related to personal care or household activities	Self-developed close-ended question	T0	
Burdens in the pandemic	Self-developed close-ended question	T2	
**Functional impairments**			
Difficulty in seeing	From EHIS [37]	T0	
Difficulty in hearing	From EHIS [37]	T0	
Difficulty in mobility (walking, climbing stairs)	From EHIS [37]	T0 – T3	x (only walking)
Difficulty in biting and chewing	From EHIS [37]	T0, T3	
T0 = baseline, T1 = 4-month follow-up, T2 = 8-month follow-up, T3 = 12-month follow-up, EHIS = European Health Interview Survey, Health ABC study = Health, Aging, and Body Composition Study, MEHM = Minimum European Health Module, SF-36 = 36-item Short-Form-Health Survey, ^a^ including cancer, ^b^ some differences exist in wording			

Constructs, Measurement	Instrument/Source	Assessment timepoint	Non-responder questionnaire
**Functional impairments**			
History of falls (past 12 months)	Adapted from PROFANE [43]	T0 – T3	
Subjective memory impairment	From AgeCoDe-Study [44]	T0 – T3	x
Dizziness or vertigo (past 12 months)	Adapted from NHANES [45]	T0	
Urinary incontinence (past 12 months)	From EHIS [37]	T0	
Fecal incontinence (past 4 weeks)	Self-developed close-ended question	T0	
Shortness of breath	MRC Breathlessness Scale [46, 47]	T0	
Activity limitations	MEHM [35, 36]	T0	x
Difficulties with activities related to personal care: basal activities of daily living, ADL	According to Katz et al. [48]; from EHIS [37]	T0 – T3	
Difficulties with household activities: instrumental activities of daily living, IADL	According to Lawton and Brody [49]; from EHIS [37]	T0 – T3	
Officially recognized disability	Self-developed close-ended question	T0	
Long-term care benefits (German: Pflegegrad)	Self-developed close-ended question	T0, T2, T3	x
**Health care**			
Type of health insurance	Self-developed close-ended question	T0	
Hospital admissions (past 12 months)	From EHIS [37]	T0 – T3	
Emergency care visits (past 12 months)	Self-developed close-ended question	T0	x
Process quality of primary care	Self-developed close-ended question	T0	
Vaccination against flu (last time)	From EHIS [37]	T0	
Number of prescribed medications	Self-developed close-ended question	T0, T3	x
List of health care products	Adapted from NRW80+ [50]	T0	
Use of assistive devices, fitness trackers, wearables	Adapted from Bitkom [51]	T0	
**Psychological assessment**			
Depressive symptoms (past 2 weeks)	PHQ-2 [52]	T0 – T3	
Health related quality of life: single question on energy (past 4 weeks)	SF-12 [53, 54]	T0, T3	
Quality of life: two questions on autonomy and on satisfaction	WHOQOL-OLD [55]	T0 – T3	
with social participation			
Self-perceived current life satisfaction	Adopted from SOEP [56]	T0 – T3	
Loneliness	According to R-UCLA [57], from SHARE [58]	T0 – T3	
General self-efficacy expectancy	GSE-3 [59, 60]	T0	
Health literacy – Healthcare: two questions on appraise information and understand information	HLS-EU [61]	T0	
T0 = baseline, T1 = 4-month follow-up, T2 = 8-month follow-up, T3 = 12-month follow-up, ADL = Activities of Daily Living, AgeCoDe-Study = Ageing, Cognition and Dementia in Primary Care Patients Study, EHIS = European Health Interview Survey, GSE-3 = General Self-Efficacy Short Scale-3, HLS-EU = European Health Literacy Survey Questionnaire, IADL = Instrumental Activities of Daily Living, MEHM = Minimum European Health Module, MRC = Medical Research Council, NHANES = National Health and Nutrition Examination Survey, NRW80+ = Lebensqualität und Wohlbefinden hochaltriger Menschen in NRW, Repräsentativbefragung NRW80 (German: representative panel study on quality of life and well-being of very old adults conducted in North-Rhine Westphalia, Germany), PHQ-2 = 2-item Patient Health Questionnaire, PROFANE = Prevention of Falls Network Europe, R-UCLA = Revised UCLA Loneliness Scale, SHARE: Survey of Health, Ageing and Retirement in Europe, SF-12 = 12-item Short-Form-Health Survey, SOEP = Socio-Economic Panel, WHOQOL-OLD = World Health Organization Quality Of Life -Older Adults Module			

Constructs, Measurement	Instrument/Source	Assessment timepoint	Non-responder questionnaire
**Health behaviors**			
Health awareness: single item	HSC [62]	T0	x
Physical activity and exercise (past 3 months)	From DEGS1 [63]	T0 – T3	
Current smoking status	From DEGS1 [64]	T0	
Fruit and vegetable consumption	From EHIS [37]	T0	
Alcohol consumption (past 12 month, past 7 days)	From EHIS [37] and from SHARE [58]	T0	
Body mass index according to self-reported height in cm and weight in kg	From EHIS [37]	T0, T3	
**Social environment**			
Social support	OSS-3 [65]	T0 – T3	
Participation: List of activities	According to NHATS [66]	T0 – T3	
Informal care and burden of care	Self-developed close-ended question	T0 – T3	x (only informal care)
Frequency of leaving the house (past month)	According to NHATS [67]	T0 – T3	
Support with activities related to personal care: basal activities of daily living, ADL	From EHIS [37]	T0 – T3	
Support with household activities: instrumental activities of daily living, IADL	From EHIS [37]	T0 – T3	
**Socio-demographics**			
Month and year of birth, sex	Self-developed close-ended question and register-based information	T0 – T3	x
Marital status	Self-developed close-ended question and register-based information	T0 – T3	
Partnership	Self-developed close-ended question	T0 – T3	x
Household size	Self-developed close-ended question	T0	
Type of housing	Self-developed close-ended question	T0, T3	
Education	Self-developed close-ended question	T0	x (only school education)
Country of birth	Self-developed close-ended question and register-based information	T0	
Net household income	Self-developed close-ended question	T0	
Financial constraints (Inability to make ends meet)	From EU-SILC [68, 69]	T0, T3	
T0 = baseline, T1 = 4-month follow-up, T2 = 8-month follow-up, T3 = 12-month follow-up, ADL = Activities of Daily Living, DEGS1 = German health interview and examination survey for adults (2008 – 2011), EHIS = European Health Interview Survey, EU-SILC = EU Statistics on Income and Living Conditions, HSC = Health Consciousness Scale, IADL = Instrumental Activities of Daily Living, NHATS = National Health and Aging Trends Study, OSS-3 = Oslo-3 Social Support Scale, SHARE: Survey of Health, Ageing and Retirement in Europe			

**Table 2:** Components of the home visit examination at month 12 follow-up Source: Gesundheit 65+

Component	Device	Measurement
**Anthropometric measures**		
Height	Portable Stadiometer Seca 213	Height in cm, accuracy of measurement: 0.1 cm
Weight	Personal scale Seca 208	Weight in kilos, accuracy of measurement: 0.1 kg
Calf circumference	Ergonomic measurement tape (Medical product), Seca 201	Circumference in cm on left calf, accuracy of measurement: 0.1 cm
**Blood pressure and heart rate**		
Resting blood pressure	Mobil-O-Graph^®^, IEM GmbH	Standardized protocol, three automated measurements in a sitting position after resting
**Physical function**		
Isometric hand grip strength	Smedley dynamometer, Scandidact, Denmark, 100 kg	Maximum grip strength achieved in four examinations alternating right and left, accuracy of measurement: 0.5 kg
**Cognitive function**		
Excecutive function	Letter Digit Substitution Test [70]	Measuring the number of digits correctly substituted within 60s
Verbal episodic memory	Word list from the German language version of consortium to establish a registry for Alzheimer’s disease [CERAD, 71]	Measuring the number of correctly recalled words per trial (Trial 1 – 3 immediate recall, Trial 4 delayed recall)
**Medication use**		
Current medication use (past 7 days)	„Arzneimittel-Erfassungs-Datenbank“, AmEDa, Medication Recording Database	On-site medication review via a computer-assisted personal interview and automated barcoding
**Statutory health insurance data**		
Personal health insurance number	Card reader for electronic health cards; software for asymmetrical encryption of personal health insurance number	Linkage to health insurance records such as diagnosis codes and drug prescription up to four years prior to examination
